# Estimation of an inter-rater intra-class correlation coefficient that overcomes common assumption violations in the assessment of health measurement scales

**DOI:** 10.1186/s12874-018-0550-6

**Published:** 2018-09-12

**Authors:** Carly A. Bobak, Paul J. Barr, A. James O’Malley

**Affiliations:** 1 Department of Quantitative Biomedical Sciences, Geisel School of Medicine, Dartmouth College, 1 Rope Ferry Road, Hanover, 03755 NH USA; 2 The Dartmouth Institute, Geisel School of Medicine, Dartmouth College, 1 Rope Ferry Road, Hanover, 03755 NH USA

**Keywords:** Bayesian analysis, Hierarchical regression, Variance function modelling, Reliability, ICC, Shared decision making, Observer OPTION^5^

## Abstract

**Background:**

Intraclass correlation coefficients (ICC) are recommended for the assessment of the reliability of measurement scales. However, the ICC is subject to a variety of statistical assumptions such as normality and stable variance, which are rarely considered in health applications.

**Methods:**

A Bayesian approach using hierarchical regression and variance-function modeling is proposed to estimate the ICC with emphasis on accounting for heterogeneous variances across a measurement scale. As an application, we review the implementation of using an ICC to evaluate the reliability of Observer OPTION^5^, an instrument which used trained raters to evaluate the level of Shared Decision Making between clinicians and patients. The study used two raters to evaluate recordings of 311 clinical encounters across three studies to evaluate the impact of using a Personal Decision Aid over usual care. We particularly focus on deriving an estimate for the ICC when multiple studies are being considered as part of the data.

**Results:**

The results demonstrate that ICC varies substantially across studies and patient-physician encounters within studies. Using the new framework we developed, the study-specific ICCs were estimated to be 0.821, 0.295, and 0.644. If the within- and between-encounter variances were assumed to be the same across studies, the estimated within-study ICC was 0.609. If heteroscedasticity is not properly adjusted for, the within-study ICC estimate was inflated to be as high as 0.640. Finally, if the data were pooled across studies without accounting for the variability between studies then ICC estimates were further inflated by approximately 0.02 while formerly allowing for between study variation in the ICC inflated its estimated value by approximately 0.066 to 0.072 depending on the model.

**Conclusion:**

We demonstrated that misuse of the ICC statistics under common assumption violations leads to misleading and likely inflated estimates of interrater reliability. A statistical analysis that overcomes these violations by expanding the standard statistical model to account for them leads to estimates that are a better reflection of a measurement scale’s reliability while maintaining ease of interpretation. Bayesian methods are particularly well suited to estimating the expanded statistical model.

## Background

R. A. Fisher first introduced the concept of an intraclass correlation coefficient (ICC) in his 1921 paper examining the familial resemblance between siblings [1]. Since then, it has become an important measurement used in the fields of psychology, genetic linkage, heritability, sensitivity analysis, study design, DNA micro array analysis, and health measurement scales [2–11]. The ICC is a measure of reliability, specifically the reliability of two different raters to measure subjects similarly [12, 13]. Inter-rater reliability is important as it demonstrates that a scale is robust to changes in raters. Hence, scales with high inter-rater reliability are less prone to measurement error such as caused by variation in human judgement [13].

In the area of health measurement scales, the ICC has been integrated into the Consensus-based Standards for the selection of the health status measurement instruments (COSMIN) check list. This checklist was developed to assess the methodological quality of studies based on measurement attributes. One of the major boxes on the COSMIN check list is reliability, where it is recommended that the ICC be used as a measurement of inter-rater reliability [9, 10]. One of the driving factors of the uptake of the ICC in many fields is its ease of interpretation [9, 10]. The ICC is a value between 0 and 1, where values below 0.5 indicate poor reliability, between 0.5 and 0.75 moderate reliability, between 0.75 and 0.9 good reliability, and any value above 0.9 indicates excellent reliability [14].

However, it has been established that the ICC is subject to a variety of methodological issues [1, 15–22]. These include a sensitivity to assumptions regarding normality and homogeneous variance, as well as having a negatively biased maximum likelihood estimator and least squares estimator [1, 18, 20–22]. A variety of methods have been proposed to address these concerns. Various variance-stabilizing transformations have been proposed [1, 19], as well as bias-corrected estimators [22, 23]. There are few factors which limit the uptake of such methods. First, guidance of how to properly implement the ICC is not communicated clearly, particularly to non-statisticians. Secondly, ease of interpretation is of utmost importance with the ICC measure, and while transformations either pre-analysis or internal to a model can correct for heterogeneity in the variance of the measurement across its true value or across other variables, they do so at the cost of interpretability. This has led to problematic misuses of ICCs in a variety of disciplines, including the evaluation of health measurement scales. Herein we formulate solutions to these problems using the evaluation of an instrument for measuring shared decision making (SDM) for illustration.

### Case study background: health measurement scales and observer OPTION^5^

Employing shared decision making between clinicians and patients has been linked to improvements in patient satisfaction, reduced decisional regret, and emerging evidence of improved treatment engagement [24]. Shared decision making (SDM) can be defined as a process by which patients and clinicians make decisions together, accounting for patient preferences and values in light of the best available evidence for treatment options [25].

Measuring the quality of shared decision making implementations in clinical settings is a challenging task [26]. Patient reported measures are common, but vary in length and quality of psychometric properties. They may also be prone to biases, such as “halo” effects leading to ceiling effects in measurement [27]. Observer measures, where trained raters evaluate shared decision making may be more accurate [28].

The Observer OPTION^5^ tool is a proposed improvement of the Observer OPTION^12^ tool [29, 30]. The Observer OPTION^12^ tool has been well-established for measuring shared decision making practices but has had mixed inter-rater reliability. It has been criticized for not placing enough emphasis on the patient’s role in the SDM process [29, 30]. The Observer OPTION^5^ instrument aims to ameliorate these shortcomings.

The Observer OPTION^5^ tool is tightly focused on the idea of a collaborative deliberation model [29–31]. It’s a five item tool which produces a score between 0 and 20, which can be rescaled to 0 and 100 for interpretability. The patients rate the clinicians interactions in each of the five areas, giving a score between 0 and 4, where 0 is no effort and 4 is exemplary effort [29, 30]. The five items are as follows:

1. The clinician indicates to the patient that there are treatment options,
2. The clinician aids in the informing and deliberating process for the patient,
3. The clinician explores the pros and cons of the treatment options with the patient,
4. The clinician attempts to ascertain the patient’s preferences in response to potential treatments discussed,
5. The clinician attempts to integrate the patient’s preferences in finalizing the treatment plan.

For a measure of an instrument’s reliability to have meaning, there ought to be a standard population against which to assess the accuracy or consistency of the rater scores across encounters. However, in the case of health measurement scales, data is often pooled from multiple studies leading to a “wild west” with no standard population. Indeed, in the SDM application, encounters can occur across various institutions and in a variety of settings. We argue that the traditional calculation of the ICC, which relies on the assumption that the variance between encounters is reasonably homogeneous, is too inflexible [1, 15–22]. As well, the common implementation of a bounded scale, or a scale bounded from below, in health measurement scales often leads to heteroscedasticity. We present a measure of ICC and method for estimating it that generalizes the incumbent approach to account for both heterogeneous data and heteroscedastic variances.

To allow an ICC that caters to multiple contexts to be estimated using a general strategy, we develop a Bayesian model and computational procedure. A desirable feature of Bayesian computations is that they avoid the reliance on analytic approximations when computing estimates. This is particularly pertinent in models that include variance functions or other terms that introduce parameters in nonlinear forms, place constraints on parameters, or estimate nonlinear functions of parameters such as ICCs. It is known that Bayesian estimates can be more precise than their frequentist counterparts, especially when prior information is informative [32]. By illustrating our approach on an evaluation of the increasingly popular Observer OPTION^5^ tool, we hope to catalyze the adoption of more meaningful and informative computations of ICC across all health measurement scale applications.

While we use OPTION^5^ as a running example, the proposed methodology applies to any data collected on a bounded response scale for which the agreement between raters is sought.

The remainder of this paper is organized as follows. We provide a brief background regarding the classical form of the ICC and illustrate how it can be over-estimated when between study variability is large in the “Methods” section. In the “Case study design” section we give an overview of the study conducted to assess the Observer OPTION^5^ tool and reveal the areas of statistical misuse that compromise the measures of ICC that have been previously reported. In the “Bayesian framework” section we propose the Bayesian model that estimates an ICC which incorporates data from heterogeneous populations and allows the variance of the measurements to be heteroscedastic. The “Evaluation of Bayesian Estimation” section details our process for computing our estimates, as well as three different scenarios we used to compare the estimates of our ICC. Our results from the original study, as well as our three scenarios are presented in the “Results” section. A brief discussion of these results and their impact on future research in health measurement scales is included in the “Discussion” section.

## Methods

The ICC is mathematically defined as:

1 $$ ICC=\frac{\sigma_{b}^{2}}{\sigma_{b}^{2}+\sigma_{w}^{2}}=\left(1+\frac{\sigma_{w}^{2}}{\sigma_{b}^{2}} \right)^{-1}   $$

where $\sigma _{b}^{2}$ is the variance between encounters, and $\sigma _{w}^{2}$ is the variance of the raters within encounters. Similarly, inter-rater reliability with *k* raters is denoted as:

2 $$ R=\frac{\sigma_{b}^{2}}{\sigma_{b}^{2}+\frac{\sigma_{w}^{2}}{k}}=\left (1+\frac{\sigma_{w}^{2}}{k\sigma_{b}^{2}} \right)^{-1}   $$

Notice that (1) and (2) increase as $\sigma _{b}^{2}$, the variance between encounters, increases. This can be a potential flaw in the calculation of the ICC, as the measure is artificially inflated when the variance between different patient encounters is larger than will occur in the intended application of the instrument. For example, by pooling data from diverse study subjects, a measure will accurately discriminate between a greater proportion of subjects, inflating the ICC or reliability.

### Bayesian framework

Let *h*=1,...,*M*, *i*=1,...,*N*_*h*_, and *j*=1,…,*R* be our indices for the study, the patient-physician encounter, and the rater. In the OPTION^5^ analysis the number of studies is *M*=3, the number of encounters within the three studies are (*N*_1_=201,*N*_2_=72,*N*_3_=38), and the number of raters is *R*=2 although the methodology applies to all values of these. Let *Y* denote the OPTION^5^ score divided by 100 (for ease of interpretation), *θ* the true amount of shared decision making, and *X* indicate the use of a PDA. Although the effect of *X* is of interest to this field, our objective is to adjust for it’s effect so as to ensure that the evaluations of ICC are meaningful. Our statistical model is

3 $$ Y_{hij}|\theta_{hi},X_{hi}\sim\text{ Normal}\left(\mu_{hij},v_{hi}^{2}\right)I(0,1)  $$

where

4 $$ I\{y_{hij}\in(0,1)\}   $$

restricts the probability distribution of the measured amount of SDM to the interval 0 to 1, and

5 $$\begin{array}{*{20}l} \mu_{hij}&=\theta_{hi}+\beta_{1}(j-1.5)+\beta_{2}(X_{hi}-\bar{X}) \end{array} $$

6 $$\begin{array}{*{20}l} v^{2}_{hi}&=\sigma_{h}^{2}\theta_{hi}(1-\theta_{hi})  \end{array} $$

with $\bar {X}$ denoting the sample mean value of *X*. The dependence of $v_{hi}^{2}$ on *θ*_*hi*_ implies that the ICC depends on the true amount of SDM in the encounter; its mathematical expression is referred to as a variance function.

We view the encounters as a random sample from a large population of possible encounters about which we wish to make inferences. The sampling of the encounters and the sampling variability in them is represented mathematically as

7 $$ \theta_{hi} \vert \text{study}\sim \text{ Normal}\left(\gamma_{h}, \tau_{h}^{2}\right) I(0,1)  $$

where *I*, defined in (4), restricts the possible amount of SDM to be a proportion (0 to 1). The specification for *θ*_*hi*_ depends on parameters which are indexed by *h*, giving each study its own mean and variance. We set our prior distributions as follows:

$$\begin{array}{*{20}l} \gamma_{h}&\sim\text{ Normal}\left(\beta_{0},\omega^{2}\right)\\ \beta_{k}&\sim\text{ Normal}\left(b_{0}I(k=0),B^{2}\right),\hspace{0.5 cm} k=0,1,2\\ \sigma_{h}^{-2}&\sim\text{ Gamma}(v_{1},v_{1})\\ \tau_{h}^{-2}&\sim \text{ Gamma}(v_{2},v_{2})\\ \omega^{-2}&\sim \text{ Gamma}(v_{3},v_{3}) \end{array} $$

The choice of normal and gamma distributions for the regression (mean or location) and the variance (scale) parameters is common in practice as the conditional posterior distributions of each parameter conditional on the remaining parameters and the data are also normal and gamma distributions. This simplifies model estimation and computation.

The desire for the prior distribution to impart virtually no information onto the analysis is accomplished by specifying distributions with very large variances for the model parameters. As a consequence, the data are solely responsible for estimating the model. In this application we set *b*_0_=0.4, *B*^2^=10, and *v*_*l*_=10^−3^ for *l*=1,2,3. Note that parameters such as *θ*_*hi*_ that have restricted ranges may be assigned prior distributions with almost no mass within the allowable range if the density is not truncated. If the allowed range is a region over which the unrestricted distribution is essentially flat, then the truncated distribution will be close to uniform - essentially assuming that all allowable values of the parameter are equally likely. As well, parameters may have values such that the mean of the unrestricted distribution is outside the allowed range, and the truncated distribution will still be well-defined. Although the inverse-Gamma prior distributions assumed here for the variance parameters have been shown to yield undesirable results in some applications [33], we found that they were well suited to our case study in the sense that the results were quite robust to the prior distribution parameters. For example, the results with *v*_*l*_=10^−2^ for *l*=1,2,3 were numerically almost identical to those with *v*_*l*_=10^−3^ for *l*=1,2,3. We attribute this result to the fact that in our case study the scale of the data has a finite range, which prevents the tails of a prior distribution from having a substantial impact on the posterior.

Under the above model, the ICC for an encounter in study *h* with SDM of *θ*^∗^ is given by

8 $$ ICC_{h}(\theta^{*})=\frac{\tau_{h}^{2}}{\tau_{h}^{2}+\sigma_{h}^{2}\theta^{*}(1-\theta^{*})}  $$

Two salient features are evident in Eq. (8). Firstly, the within (*σ*^2^) and between (*τ*^2^) encounter variance and scale parameters depend on the index for study. Therefore, the ICC is study specific. Secondly, the within-encounter scale parameter is multiplied by *θ*^∗^(1−*θ*^∗^), which crucially allows for the ability of raters to agree, or rate consistently, to depend on the actual amount of SDM. Because it is easier to distinguish cases against a baseline level of a trait close to 0% or 100% than cases in which the trait is about 50% present (this is seen from the fact that the variability of a restricted scale is greatest around its middle point), the involvement of the binomial variance form *θ*^∗^(1−*θ*^∗^) makes intuitive sense.

In practice, one may choose a value of *θ*^∗^ that has particular meaning or relevance to the application at which to compute the ICC. If multiple values of *θ*^∗^ are important (e.g., the baseline levels for various population strata) a separate ICC can be reported for each of them. Alternatively, or additionally, one may also choose to average over a population of values of *θ*^∗^. For example, if we expect the population of patient-physician encounters on which the instrument will be applied to be described by the probability distribution,

$$\begin{array}{*{20}l} \theta^{*} &\sim \pi(\theta^{*}) = \text{ Normal}\left(\gamma_{h}, \tau_{h}^{2}\right)I(0,1), \end{array} $$

it follows that the population average ICC, given by

9 $$ {}{ICC}_{h}=\tau_{h}^{2} \int_{0}^{1} \left (\tau_{h}^{2} + \sigma_{h}^{2}\theta^{*}(1-\theta^{*})\right)^{-1}\pi(\theta^{*}) \hspace{0.5cm} d\theta^{*},   $$

should be computed. The evaluation of multiple measures of ICC yields a much more informative profile of an instrument’s performance than the presently used single number summary derived under overly restrictive assumptions. This function is designed in such a way that the user directly specifies a distribution for *θ*^∗^ to maintain flexibility in the calculation of the ICC. This distribution can be specified with known parameters to avoid integration over the hyper parameters *γ*_*h*_ and *τ*_*h*_ for simplicity. Alternatively, the user could assume a hierarchical prior where integration over these parameters would also be necessary.

The ICC can also be defined for a scenario where encounters are pooled across studies. Assuming an equal probability of selecting an encounter from each study, the marginal variance across these encounters is $\omega ^{2} + \bar \tau ^{2} + \bar {\sigma }^{2}\theta ^{*}(1-\theta ^{*})$ (a more general expression may be substituted if the study selection probabilities are unequal). Hence, the corresponding measure of ICC is given by

10 $$ {ICC}_{\text{Marg}}(\theta^{*})=\frac{\omega^{2}+\bar{\tau}^{2}}{\omega^{2}+\bar{\tau}^{2}+\bar{\sigma}^{2}\theta^{*}(1-\theta^{*})}   $$

Typically, one would see

11 $$ {ICC}_{\text{Marg}}(\theta^{*}) \geq \overline{ICC} (\theta^{*})  $$

Although the pooled or marginal ICC is well-defined under a specified model for sampling encounters from the individual studies, if the intended use of the instrument is to compare encounters across a homogeneous population of subjects (e.g., the reference population for a single study) then ICC _*Marg*_(*θ*^∗^) makes the instrument look better in a meaningless way as it overstates the heterogeneity between the subjects compared to the heterogeneity between the individuals in the population that the instrument will be used to compare or discriminate between in actual practice.

Summarizing the above, the three forms of ICC are seen to be components of a two-dimensional family of measures of ICC defined under the full statistical model we developed to account for the intricacies of the data. The dimensions are: 1) whether or not the ICC is specific to a particular level of the quantity being studied versus averaging over a distribution of values of that quantity; 2) whether or not variability between studies is included in the between encounter variance (which corresponds to whether or not it is desired for the instrument to discriminate between encounters from different studies in practice). Combining these two dimensions, there are four general types of ICC that are available under the general approach we have proposed.

### Evaluation of Bayesian Estimation

All analyses of the Observer OPTION^5^ data were conducted in R [34]. Markov Chain Monte-Carlo (MCMC) simulation for Bayesian estimation was implemented using Just Another Gibbs Sampler (JAGS) and integrated with pre- and post-processing using the R package ‘rjags’ [35, 36]. Three Bayesian models for three separate ICC scenarios are compared. The first is the full model, which separately calculates the posterior variance and ICC for each study. The second restricts the variances to be homogeneous across the three studies. The third ignores the issue of within-study heteroscedasticity in the variability of raters’ assessments of the amount of SDM. Posterior distributions are summarized by their median and 95% symmetric credible interval (2.5th and 97.5th percentiles).

### Case study design

Our main study of interest can be found in [30]. Data was collected from two previous studies, the Chest Pain Choice trial (Study 1) and the Osteroporosis Choice Randomized trial (Studies 2 and 3) [37, 38]. Both trials randomly assigned patients to either receive an intervention of use of a Personal Decision Aid (PDA), or receive usual care [37, 38]. The Osteoporosis Choice Randomized trial contains a subgroup of participants who used the World Health Organization’s Fracture Risk Assessment Tool (FRAX®;) [38]. For the purposes of our analysis, we consider patients who used FRAX®; as a separate study group (Study 3). The Chest Pain Choice trial recruited participants from St. Mary’s Hospital Mayo Clinic in Rochester, MN while the Osteoporosis Choice Randomized trial recruited from 10 general care and primary care practices in the Rochester, MN area [37, 38].

Audio-visual recordings of the patient-clinician encounters took place and two-raters independently assessed the recording of each patient-physician encounter across these three clinical studies of decision-aids using the Observer OPTION^5^ SDM tool. A total of 311 clinical encounters were included in the study Table 1 summarizes these encounters across the three studies of interest. The overall Observer OPTION^5^ score was calculated for each encounter and rater [30]. The goal of the following analysis is to determine the concordance of the two raters despite the heterogeneity of the study groups and inherent heteroscedasticity.

**Table 1:** Encounters from the three randomized studies which compared the impact of PDAs to standard care

Study	PDA (n)	Usual care (n)	Total
1	101	100	201
2	37	35	72
3	13	25	38
Total	151	160	311

In this particular case, the recorded encounters from all three studies were re-rated by the same two raters. Hence, we assume that the differences across the studies are due to the differences in populations and imposed interventions across each study. In many cases, there would also be heterogeneity across raters of studies, leading to even greater between-study heterogeneity than is observed in this case.

## Results

**Fig. 1:**
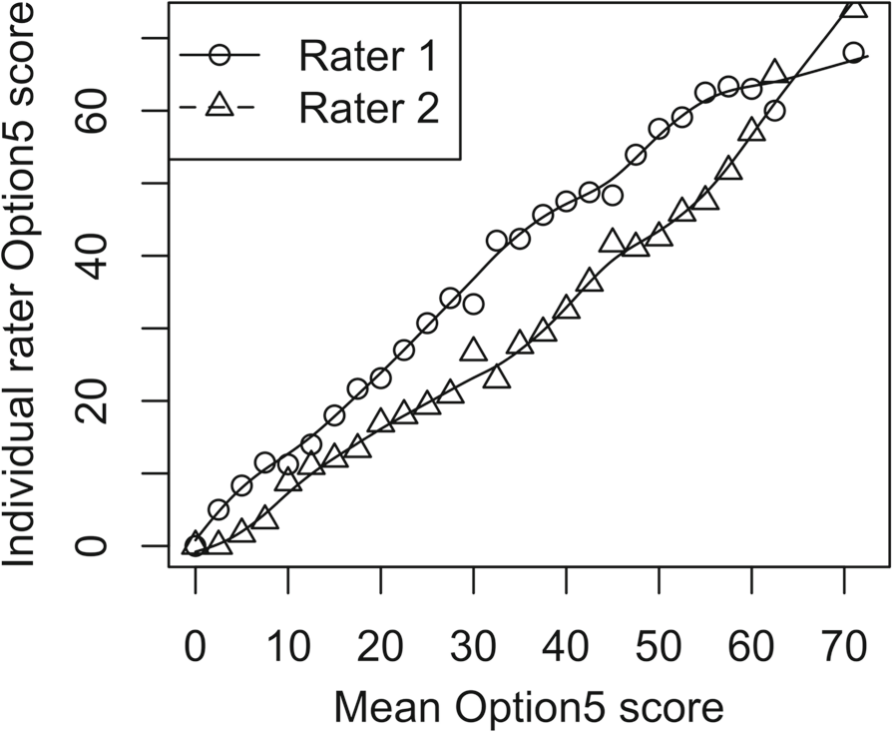
Comparison of Observer OPTION^5^ scores between raters. The individual rater score is shown on the y-axis and the mean OPTION^5^ score is shown on the x-axis

The results from the rater’s independent assessment of SDM using Observer OPTION^5^ are shown in Fig. 1. In general, Rater 1 consistently scored encounters higher than Rater 2.

**Fig. 2:**
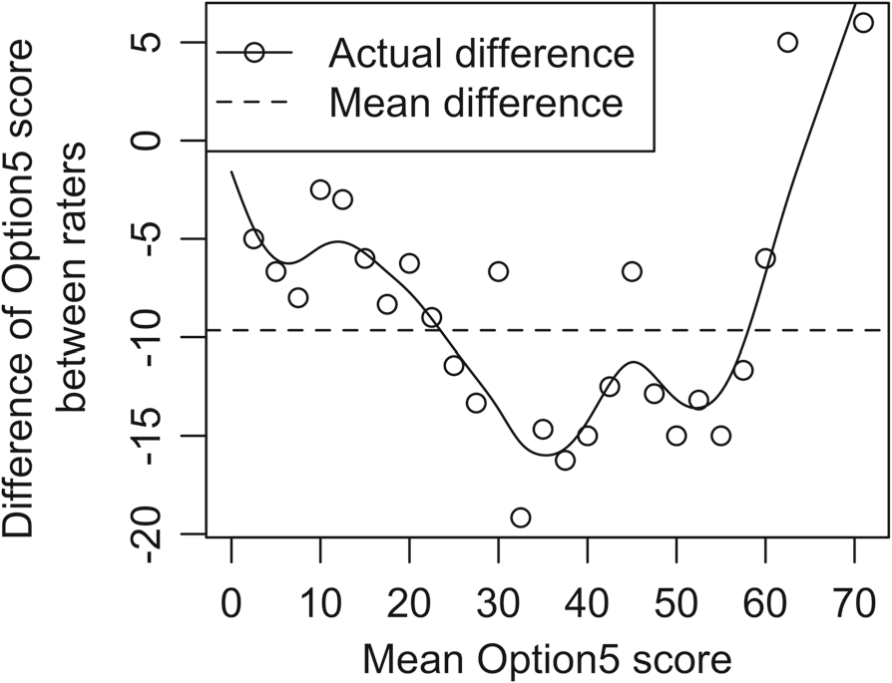
Actual difference of Observer OPTION^5^ between raters over the mean OPTION^5^ score. While the average difference is slightly less than 10, this difference varies greatly across the mean score, demonstrating non-constant variance

Figure 2 shows the actual difference of Observer OPTION^5^ scores as a function of the mean Observer OPTION^5^ score for each encounter. The mean difference across the encounters is approximately 10 points, although over the range of 30–60 the sample differences were consistently on the order of 12 to 16 and as high as 19.

**Fig. 3:**
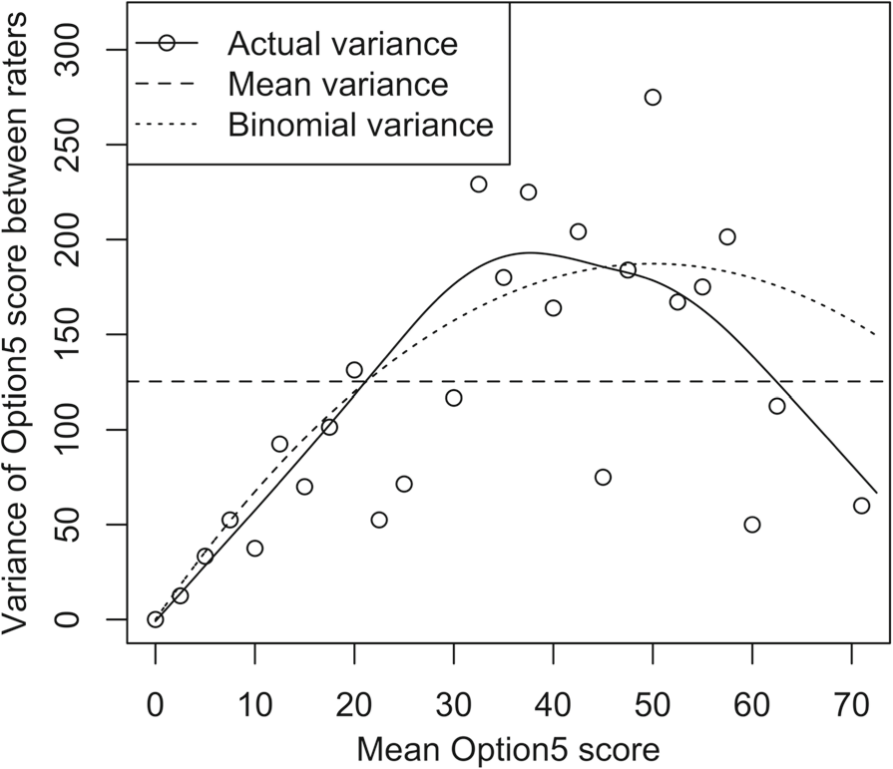
Empirical variance of scores Compares the mean variance, binomial variance, and the observed variance (using a smoothing spline with 10 degrees of freedom) of Observer OPTION^5^ score. Highlights the heteroscedasticity of the variance as a function of the mean

Figure 3 shows the empirical variance functions. The observed variance connected with a smoothing spline with 10 degrees-of-freedom is shown in the solid line, while the mean and binomial variance functions are shown in dashed lines. It is clearly demonstrated that the variance is heteroscedastic and the sample mean variance across the encounters would yield a poor representation of the data. The binomial variance function performs better, suggesting that (6) may be an adequate model for the dependence of the variability of the raters’ scores on the amount of SDM in the encounter.

A virtue of the Bayesian approach is that it avoids analytical approximations, even in complicated situations. The ICC is an example of a nonlinear function of parameters whose exact estimate and other inferences require variable transformation and high dimension integration to obtain the marginal posterior of the ICC. This happens automatically when Monte Carlo averages are evaluated over draws of parameters from the joint posterior distribution without requiring complicated mathematics to make accurate approximations. Hence, the joint posterior is used implicitly by the user.

**Fig. 4:**
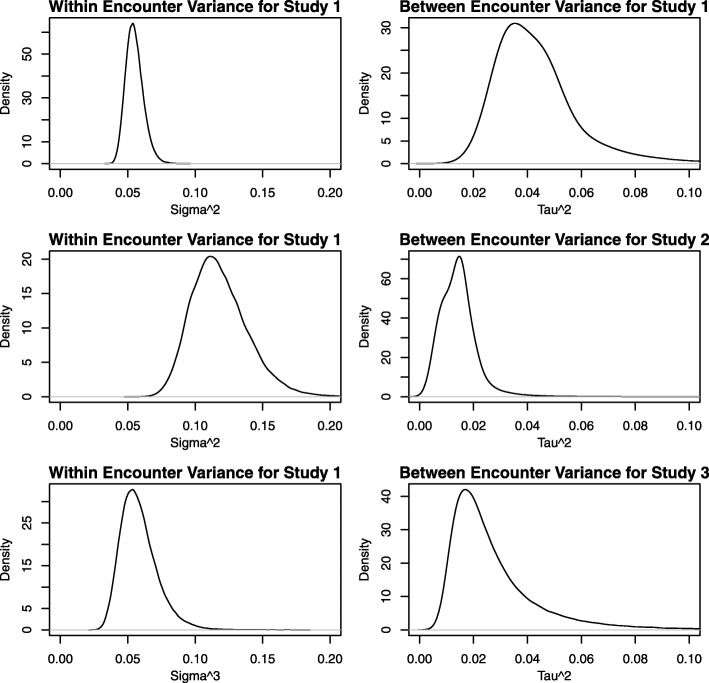
A comparison of the posterior distribution of the key parameters underlying the ICC between the within-encounter variance and the between-encounter (but within study) variance across the three studies

**Fig. 5:**
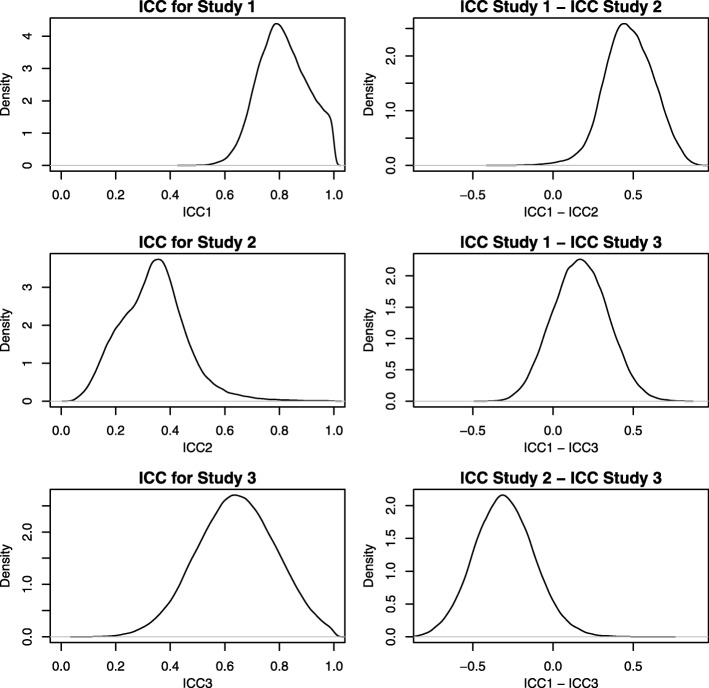
Posterior distributions of the ICCs for each study, and the difference in the ICC for each pair of studies

To further illustrate the utility of our approach, we have produced plots of the posterior distribution of the key parameters underlying the ICC, the within-encounter variance and the between-encounter (within study) variance, for each study in Fig. 4. We also have made plots of the ICCs for each study and the difference in the ICC for each pair of studies in Fig. 5. In addition, we have also summarized the differences in the ICC between studies in terms of the posterior mean, posterior median, the 2.5 and 97.5 quantiles, and the posterior probability that the difference exceeds 0 in Table 2. Together, these figures and summary statistics provide a detailed description of the heterogeneity in the reliability of the measurement properties for each study and the statistical significance of differences between them. Such inferences are exact (to the numerical precision of the number of iterations we ran the MCMC procedure) and are trivial to obtain using Bayesian computation whereas more laborious and specialized calculations would be needed with frequentist calculation.

**Table 2:** The differences in the ICC between studies in terms of the posterior mean, median, the 2.5 and 97.5 quantiles, and the posterior probability that the difference exceeds 0

Paired difference	2.50%	Median	Mean	97.50%	*p*-value
Study 1 - Study 2	0.166	0.472	0.473	0.764	0.995
Study 1 - Study 3	-0.155	0.170	0.171	0.508	0.835
Study 2 - Study 3	-0.659	-0.306	-0.302	0.078	0.056

The hyperparameters for completing the specification of the Bayesian model are set to the values given above Eq. (8). Results from a full model, which takes the heterogeneity of the different studies into account are shown in Table 3. Summary ICC estimates for Study 1, 2, and 3 were computed for a hypothetical new patient from the population of patients represented in each study (this corresponds to the population average ICC computed in Eq. 9). Note that the variance estimates are scaled to represent the rescaling of the OPTION^5^ score from (0,100) to (0,1). The resulting posterior means of the ICC were 0.821, 0.295, and 0.644 respectively. The estimate for Study 2 is particularly low in comparison to Study 1 (the posterior probability that study 1 has a higher ICC than study 2 =0.995) and Study 3 (the posterior probability that study 3 has a higher ICC than study 2 =0.944), demonstrating the extent of the heterogeneity between each study and how the ICC appraises very different impressions of the performance of Observer OPTION^5^ across the studies due to differences in the variability of SDM in the encounters it is trying to discriminate between. The credible intervals associated with the ICC estimates are quite wide due to the relative small sample sizes in two of the studies and the fact that there are only three studies to inform the between-study variance component, *ω*.

**Table 3:** Full model results from Bayesian Framework*

Term	Posterior summary		
	Median	2.5%	97.5%
*β*[0]	0.145	-0.087	0.490
*β*[Rater]	-0.061	-0.073	-0.051
*β*[Decision-aid]	0.239	0.214	0.270
(*σ*/100)^2^[Study 1]	0.054	0.044	0.070
(*σ*/100)^2^[Study 2]	0.117	0.084	0.168
(*σ*/100)^2^[Study 3]	0.056	0.037	0.090
*τ*^2^[Study 1]	0.043	0.024	0.097
*τ*^2^[Study 2]	0.011	0.004	0.034
*τ*^2^[Study 3]	0.023	0.009	0.078
*ω*	0.029	0.003	0.717
ICC[Study 1]	0.821	0.655	0.985
ICCStudy 2]	0.295	0.119	0.628
ICC[Study 3]	0.644	0.359	0.919

^*^Here, the ICC for each study refers to the conventional within-study ICC (see Eq. 8) averaged over a population of encounters (Eq. 9)

The above results are further emphasized when we compare them to the results for the model with pooled study estimates and homogeneous variances using the same Bernoulli variance function for all three studies in Table 4. The pooled ICC estimate is 0.609, a notable reduction for Study 1 and severely inflated compared to the separate estimate for Study 2. An interesting observation is that the estimate of the between study variance, *ω*, is substantially less when study-specific estimates of the within- and between-encounter variances are not permitted (compare Table 4 to Table 3), illustrating how variation can be erroneously partitioned between levels of a model if there is substantial heterogeneity between the studies. We also tested a scenario for a pooled ICC estimate without accounting for the dependence of the within-encounter variance, hence assuming a constance variance across studies, on the true level of SDM in the encounter. We clearly see evidence of an inflated ICC with the estimated ICC of 0.640 (Table 5) substantially exceeding the pooled ICC estimate. If the study effects were ignored altogether (i.e., complete pooling of the data) then ICC estimates were further inflated by approximately 0.02 (model not presented) while incorporating between study variation in the ICC via Eq. (10) yields estimates 0.072 or 0.066 greater depending on whether heteroscedasticity was accounted (Table 3) or ignored (Table 4).

**Table 4:** Results for homogeneous variance using a Bernoulli variance function to capture heteroscedastic variance

Term	Posterior summary		
	Median	2.5%	97.5%
*β*[0]	0.317	0.203	0.449
*β*[Rater]	-0.088	-0.102	-0.074
*β*[Decision-aid]	0.250	0.217	0.281
(*σ*/100)^2^	0.041	0.035	0.048
*τ*	0.015	0.011	0.019
*ω*	0.004	0.001	0.092
ICC	0.609	0.520	0.745
ICCb ^∗^	0.681	0.568	0.935

^*^ICCb denotes the ICC for the case when encounters are pooled across studies (see Eq. 10) whereas ICC is the conventional within-study ICC (see 8). In both cases the ICC is averaged over a population of encounters, as in 9

**Table 5:** Results for homogeneous variance using a constant variance function

Term	Posterior summary		
	Median	2.5%	97.5%
*β*[0]	0.319	0.206	0.451
*β*[Rater]	-0.097	-0.111	-0.083
*β*[Decision-aid]	0.278	0.248	0.309
(*σ*/100)^2^	0.008	0.007	0.009
*τ*	0.014	0.011	0.017
*ω*	0.004	0.001	0.091
ICC	0.640	0.568	0.702
ICCb ^∗^	0.706	0.614	0.930

^*^ICCb denotes the ICC for the case when encounters are pooled across studies (see Eq. 10) whereas ICC is the conventional within-study ICC (see 8). In both cases the ICC is averaged over a population of encounters, as in 9

**Fig. 6:**
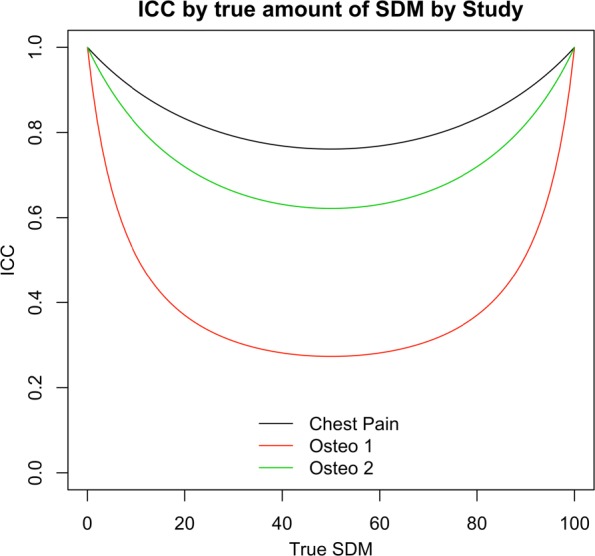
Direct analysis of ICC as a function of level of agreement Relationship of ICC to the true amount of shared decision making (SDM) in an encounter and heterogeneity of reliability of measurements across studies. The ICC is higher at the ends of the scale than at the center where the variability under the binomial variance function of rater scores on the same encounter is greatest and the difference in the reliability of measurements across the studies is substantial

Figure 6 illustrates the dependence of the ICC on the true amount of SDM in an encounter and the study in which the encountered occurred. The ICC trajectory lines for each study were constructed by evaluating the posterior mean of the ICC defined in (8) at 101 values of SDM (*θ*^∗^) evenly spaced from 0 to 1. Due to the concave shape of the variance function, encounters are easier to discriminate when the mean is closer to 0 or 100 than to 50 with the difference quite substantial. The contrasting level of ICC across the three studies further emphasizes their heterogeneity.

## Discussion

According to the COSMIN checklists, assessing the inter-rater reliability of instruments is essential when proposing novel health measurement scales [9, 10]. These guidelines recommend assessing the ICC to examine the reliability of proposed measures, but only briefly allude to the limitations of broadly applying ICC (or reliability) estimates. As a result, the mass use of the ICC in the field of health measurement has lead to a variety of studies which may have miss-stated the reliability of new instruments from ignoring heteroscedasticity. For instance, Scholl *et al* review a collection of instruments with restricted scales that calculate ICC or inter-rater reliability many of whom do not account for the dependence of the variability of measurements on the value of the quantity being measured [28]. As well, there are many other studies of instruments with restricted scales which have given no indication that the assumption of homogenous variance has been met [39–41]. All of these studies are candidates for having miss-stated the ICC, and would benefit from implementing the framework proposed here. Furthermore, ICC estimates often have great implications in experimental design considerations, particularly in terms of properly powering studies. Hence, it is extremely important to have estimates that account for heteroscedasticity and apply to the context in which the instrument is planned to be used [22].

In a published guideline for selecting and reporting ICC measures, Koo and Li discuss 10 different forms of ICCs based on the model, type of measurement whether it be the mean of k-raters or a single-raters measurement, and whether or not absolute agreement or consistency is desired [14]. The measurement we’ve proposed here is an inter-rater, inter-case discriminatory ICC and hence applies for forms of the ICC considering multiple raters and emphasizing consistency of measurements.

We demonstrated that the ICC is inflated under a homoscedastic variance assumption and that multiple studies should not be pooled in order to calculate an overall ICC for an instrument when there is substantial heterogeneity between studies. We’ve proposed a framework which is robust to heteroscedastic variance while maintaining ease of interpretation for use by clinicians and other non-statisticians. As well, the implementation of a Bayesian framework negates the issue of a biased estimate for the ICC, as Bayesian estimates do not rely on closed-form approximations and normal distribution asymptotic theory [32, 42].

Because a wide variety of appraisals of Observer OPTION^5^ are possible using these data, it is possible that overly optimistic assessments could have been published and erroneously influenced research using Observer OPTION^5^ to measure SDM (e.g., studies may be under-powered) if assumptions were not clearly described. We hope that the methodology outlined in this paper will be adopted widely and lead to correctly calibrated estimates and descriptions of ICC and, therefore, more informative profiles of instrument quality being used in important applications.

It should be noted, that while this approach will work for any case in which the measurement is bounded, it may be overkill when examining data from a single study where the outcome is tightly distributed around the middle of the scale. In that particular situation, conventional approaches for calculating the ICC should be satisfactory. In the absence of these conditions, the approach we are proposing here should be utilized.

An advantage of the framework developed in this paper is that it applies to any number of studies, raters, and encounters within studies. However, as the ICC is often calculated by non-statisticians, a potential limitation of this framework is the perceived learning curve in applying a Bayesian approach for estimation. Frequentist approaches dominate the medical literature, although it has been argued that clinicians naturally use Bayesian thinking in their everyday decision making [43]. To aid in the easy implementation of our framework, we deposited the R code used to generate our estimates in GitHub (see Availability of data and materials) along with the code we used to simulate data from the model described here to avoid potential patient data confidentiality issues.

While this paper mostly focused on the context of calculating an ICC in terms of developing measurement scales for health practitioners, this framework naturally extends into many other fields. Future work will include extending the approach to other fields of study.

## Conclusion

Despite its wide-spread use as an important measure of inter-rater reliability, there are a variety of established methodological issues that need to be considered in specifying and estimating an ICC [1, 15–22]. As it is a metric which is frequently applied by non-statisticians, there is concern that these issues are not properly being accounted for and, as a result, inflated ICC estimates are being published in the literature across a variety of fields. In this work, we propose a Bayesian framework for estimating the ICC that accounts for heteroscedastic variances and avoids relying on an estimator’s normality for inferences to be correct. A particular strength of this approach is that it yields estimates which are robust to many common errors in ICC calculation while maintaining straight-forward interpretation for researches across many fields of interest. Widespread adoption of this model-based ICC definition and allied estimation procedure would ultimately lead to more flexible and accurate representation of inter-rater reliability.

## Abbreviations

COSMIN: Consensus-based standards for the selection of health status measurement instruments
ICC: Intraclass correlation coefficient
JAGS: Just another Gibbs sampler
MCMC: Markov chain Monte-Carlo
PDA: Personal decision aid
SDM: Shared decision making
